# Real World Clinical Experience of Biosimilar G-CSF (Grastofil) for Autologous Peripheral Blood Stem Cell Mobilization: Single Center Experience in Canada Following Early Adoption

**DOI:** 10.3390/curroncol28030148

**Published:** 2021-04-22

**Authors:** Vibhuti Aggarwal, Waleed Sabry, Mohamed Elemary, Mark Bosch, Pat Danyluk, Prosanta Mondal, Julie Stakiw

**Affiliations:** 1 College of Medicine, University of Saskatchewan, Saskatoon, SK S7N 5E5, Canada; vibhuti.aggarwal@usask.ca (V.A.); waleed.sabry@saskcancer.ca (W.S.); mohamed.elemary@saskcancer.ca (M.E.); mark.bosch@saskcancer.ca (M.B.); pat.danyluk@saskcancer.ca (P.D.); 2 Saskatchewan Cancer Agency, Saskatoon, SK S7N 5E5, Canada; 3 Clinical Research Support Unit, University of Saskatchewan, Saskatoon, SK S7N 5E5, Canada; prosanta.mondal@usask.ca

**Keywords:** Grastofil 1, Biosimilar 2, Stem Cell Mobilization 3

## Abstract

*Introduction:* Granulocyte colony-stimulating factor (G-CSF) is the first line treatment for mobilization, most commonly using a regimen of daily filgrastim. The use of biosimilars can provide substantial cost savings to the health care system while delivering comparable efficacy outcomes. In 2016, the Saskatchewan Cancer Agency was a leader in Canada, instituting formulary changed from a G-CSF originator product to a cost savings alternative biosimilar for stem cell mobilization prior to autologous stem cell transplant (ASCT) and for engraftment. The purpose of this study was to investigate the clinical comparability of biosimilar G-CSF to its reference product in a real-world clinical setting and to validate use of the biosimilar in mobilization and engraftment—an indication which had been granted by extrapolation. Methods: A retrospective chart review was completed including all patients diagnosed with a hematological malignancy between 2012 and 2018 who underwent ASCT. To assess real-world outcomes across a diverse population, successful CD34+ stem cell collection was compared between patients mobilized with originator filgrastim, Neupogen, and biosimilar filgrastim, Grastofil. Additional comparisons included the number of apheresis required, time to absolute neutrophil count (ANC) engraftment, platelet engraftment, length of hospital stay, and Plerixafor use. Results: 217 patients were mobilized and transplanted during the study period. There was no statistically significant difference in success rate between patients mobilized with biosimilar filgrastim and those who had received originator G-CSF (100% vs. 92.4%, *p* = 0.075). Neither disease type, nor concurrent chemomobilization regimen resulted in a detectable difference between the two G-CSF products in successful stem cell harvest. Engraftment was highly similar between groups, as demonstrated by ANC recovery (11.6 days Neupogen vs. 11.6 days Grastofil), platelet recovery (14.0 days Neupogen vs. 14.2 days Grastofil), and total length of hospital stay (22.4 days Neupogen vs. 22.3 days Grastofil). No statistically significant difference in adjunctive use of Plerixafor^®^ was observed between Neupogen and Grastofil patients (25.9% vs. 23.4%, *p* = 0.72). Conclusion: Extrapolation of indications for biosimilars is justified. This real-world evidence builds upon registrational studies to confirm that no clinically meaningful differences were detected between originator Neupogen and biosimilar Grastofil in the setting of PBSC mobilization and engraftment post ASCT. Biosimilars are as safe and effective as originator products. Implementation across all approved indications without hesitation maximizes cost savings to the provincial system, allowing for more optimal allocation of health care resources.

## 1. Introduction

Patients diagnosed with hematologic malignancies including multiple myeloma (MM), Hodgkin’s disease, and aggressive lymphomas may receive myeloablative therapy followed by autologous stem cell transplant (ASCT) as standard first-line therapy. ASCT represents a viable curative treatment option for transplant eligible patients and peripheral blood stem cells have become the most widely used source of hematopoietic stem cells in this setting using various strategies for chemomobilization and collection [1,2]. To effectively collect hematopoietic stem cells from peripheral blood for transplant, growth factors and other pharmacologic agents are used to mobilize stem cells from the bone marrow [3,4]. Granulocyte colony-stimulating factor (G-CSF) is the first line treatment for mobilization, most commonly using a regimen of daily filgrastim (rHu-G-CSF, originator brand Neupogen^®^, AMGEN, Thousand Oaks, CA, USA) [5,6]. Filgrastim is a therapeutic biological product, a single polypeptide chain protein based on a 174-amino-acid sequence produced through recombinant DNA biotechnology. Following the patent expiry for Neupogen in 2006, several biosimilar filgrastim products have been approved by global regulatory authorities, with Grastofil^®^ (filgrastim, Apotex, North York, ON, Canada), marketed in the EU as Accofil^®^ (Accord Healthcare, North Harrow, Middlesex, UK) being the first approved biosimilar G-CSF product in Canada [7]. 

High price biologic agents challenge healthcare budgets and limit access to medicines, motivating the need for more cost-effective biosimilars. While rapid adoption can maximize cost-savings, delayed implementation of biosimilars due to physician uncertainty can substantially limit healthcare systems’ opportunity for greater realized savings. In 2016, the Saskatchewan Cancer Agency formulary effectively changed from G-CSF originator product to a cost savings alternative biosimilar for all labeled uses including stem cell mobilization prior to ASCT and for engraftment. Across Canada, however, because no efficacy data was available in this setting from registration studies nor any real-world clinical data reported on G-CSF biosimilar, physician hesitancy prompted many institutions to continue relying upon higher priced originator products. 

Since their introduction into Europe and the United States, numerous reports have indicated that clinician knowledge and attitudes towards biosimilars are barriers to implementation. Among specialty clinicians, including oncologists who regularly prescribe biologic agents, knowledge gaps related to defining original biologics and biosimilars, understanding the approval process and comparability exercise of biosimilars, and the rationale for extrapolation of indications. Additionally, clinicians were in disagreement about collaborative decision making about biosimilars between physicians and pharmacists, and disagreed about the role of patient choice in choosing biosimilars. Clinicians’ highest concerns about using biosimilars relate to availability of safety and efficacy data.

In contrast to an originator biologic, whose regulatory approval is based on pivotal clinical studies of safety and efficacy, the regulatory approval pathway for biosimilars uniquely derives from an extensive comparability exercise with the reference biologic product with significant emphasis on comparative molecular analytics, and head-to-head pharmacokinetic (PK) and pharmacodynamic (PD) evaluations to ensure no clinically meaningful differences exist between the biosimilar and its reference product [8,9]. Establishing safety and efficacy for each indication of the originator product may not be required for the approval of a biosimilar. Based on the totality of the evidence approach, a biosimilar may be granted some or all the clinical indications of its reference product label absent the need to show equivalence in confirmatory studies in each approved clinical setting [10,11]. 

Grastofil^®^ was licensed by Health Canada for all indications of the reference product based on extrapolation of clinical data in healthy adults and a single phase III efficacy and safety study in the primary prophylaxis of chemotherapy-induced neutropenia in breast cancer patients [12]. Regulatory approval was also supported by four comparative pharmacokinetic and pharmacodynamic studies that included mobilization of CD34+ stem cells in the peripheral blood analyzed as a secondary PD endpoint. Published experience using multiple biosimilar filgrastim products in the autologous transplantation setting continues to be advanced following approvals worldwide, with data from actual clinical practice indicating that biosimilar G-CSF demonstrates no clinically meaningful difference in treatment outcomes when compared to the original G-CSF [13,14,15,16,17]. Despite the lack of phase III clinical data in the setting of ASCT for regulatory approval in Canada and worldwide, biosimilar G-CSF is widely used across all labeled indications. 

While the body of real-world evidence is growing for use of biosimilars across extrapolated indications, clinician uptake remains slow. The purpose of this study was to describe the experience of the first Canadian province to implement biosimilar G-CSF and endorse use across all indications. We evaluated the clinical comparability of biosimilar G-CSF to its reference product for mobilization and engraftment outcomes in a real-world clinical setting and described results in terms of both clinical and practical endpoints, including successful stem cell harvests, adjunctive plerixafor (Mozobil^®^, Genzyme, Cambridge, MA, USA) use, number of days for apheresis, length of stay in hospital, and days to engraftment. 

## 2. Materials and Methods

A retrospective chart review of data from The Saskatchewan Cancer Agency was conducted on all patients diagnosed with a hematological malignancy, including multiple myeloma, lymphoma, amyloidosis, and other transplant eligible patients undergoing ASCT between 2012 and 2018. Inclusion of all transplant eligible patients in the survey was intended to capture the broadest real-world assessment of this center’s experience. To compare the efficacy of Neupogen and Grastofil, the primary endpoint of successful CD34+ stem cell harvest was defined as the collection of ≥4 × 10^6^ CD34+ cells/kg when collecting for two ASCT, or ≥2 × 10^6^ CD34+ cells/kg for a single planned AASCT. Success was further defined as achieving for patients who underwent ASCT included the time to absolute neutrophil count (ANC) engraftment (>0.5 × 10^9^/L for 2 days) and platelet engraftment (>20 × 10^9^/L for 2 days), in addition to length of hospital stay and time from transplant to discharge post-transplant.

The data were described for the efficacy of mobilization with both G-CSF products for various malignancies in terms of successful harvest, patients requiring Plerixafor^®^, number of days for apheresis, length of stay in hospital, and time to engraftment. This study was granted an ethics exemption by the Ethics Committee at The Saskatchewan Cancer Agency.

### Statistical Analysis

Number and percentages were reported to describe categorical variables while mean, with standard deviation (SD) and median with inter-quartile range (IQR) were provided for continuous variables. To compare continuous variables between groups, the *t*-test and Wilcoxon non-parametric tests were used for normally, non-normally distributed variables, respectively. To find an association between categorical variables, Chi-square, Fisher’s exact tests were performed as appropriate. A *p*-value of less than 0.05 (two-sided) was considered as statistically significant. We used SAS 9.4 (SAS Institute, Inc., Cary, NC, USA) to conduct all statistical analyses.

## 3. Results

The analysis included records for a total of 217 patients mobilized and transplanted by the Saskatchewan Cancer Agency between 2012 and 2018. Between 2012 and 2016, 170 patients completed mobilization with Neupogen and following the formulary switch and implementation of Grastofil in 2016, a total of 47 patients mobilized using the biosimilar were included in this analysis (Table 1). 

Patients included in the analysis were predominantly older males with hematologic malignancy. Patients mobilized with Grastofil after 2016 were slightly older than the reference cohort who had been mobilized with Neupogen. The ratio of lymphoma to multiple myeloma was consistent across groups, however the practice of chemo-mobilization was more common in the reference cohort than in the group following the implementation of Grastofil.

Across the full study period, patients with multiple myeloma/amyloidosis (*n* = 122) were either chemo-mobilized with cyclophosphamide in addition to G-CSF or received G-CSF as the sole agent for mobilization (26.2% and 73.8%, respectively). Meanwhile, patients treated for lymphomas or other hematologic diseases (listed in Table 1, *n* = 95) were chemo-mobilized in all cases, and either received cyclophosphamide-etoposide (CE) or rituximab-cytarabine (R-AraC) in addition to G-CSF. 

Overall, 204 of the 217 (94%) patients who were mobilized for ASCT achieved successful target CD34+ collection, with mean CD34+ count of 6.2 × 10^6^ CD34+ cells/kg, reflecting overall excellent leukapherisis outcomes for both cohorts. There was no statistically significant difference in success rate between patients mobilized with biosimilar filgrastim and those who had received originator G-CSF (100% vs. 92.4%, *p* = 0.075). 

In post hoc subgroup analyses, among patients with Multiple Myeloma and Amyloidosis who received G-CSF mobilization (no chemotherapy), 95.3% of patients given Neupogen^®^ (AMGEN, Thousand Oaks, CA, USA) had a successful harvest compared to 100% of Grastofil^®^ patients achieving a successful harvest (*p*-value = 0.55), indicating that the choice of G-CSF product had no clinical relevance. Patients diagnosed with lymphomas or other malignancies who received chemotherapy during mobilization demonstrated similar efficacy for stem cell mobilization with 92.1% of patients successfully harvested using the originator product, compared to 100% of patients achieving successful stem cell harvest using the biosimilar (*p* = 0.34).

The similarity between mobilization cohorts also reflected no statistically significant difference in adjunctive use of Plerixafor^®^ among patients requiring increased stem cell mobilization due to low peripheral CD34+ count the day prior to scheduled harvest (Table 2). Multiple Myeloma and amyloidosis patients who were mobilized using either Neupogen^®^ or Grastofil^®^ with no chemotherapy required Plerixafor^®^ in 21.9% and 19.2% of cases, respectively (*p* = 0.78). In patients diagnosed with lymphoma or other malignancy who received chemotherapy during mobilization, 25.0% of patients given Neupogen^®^ required Plerixafor^®^ compared to 31.6% of Grastofil^®^ patients (*p* = 0.56).

There was no statistically significant difference in requirement for >1 day of apheresis observed between patients mobilized with Neupogen^®^ versus those mobilized with Grastofil^®^ (48.8% vs. 59.6%, respectively, *p* = 0.19). In additional subgroup analysis stratified by disease type (data not shown), among patients diagnosed with plasma cell disorders, 59.4% of those mobilized with Neupogen^®^ required more than 1 apheresis day compared to 76.9% of Grastofil^®^ mobilized patients (*p* = 0.11). Due to their disease type, these patients underwent collection to the higher target of ≥4 × 106 CD34+ cells/kg. For lymphoma patients who were mobilized with G-CSF and chemotherapy and apheresed to a goal of ≥2 × 10^6^ CD34+ cells/kg, there was also no statistically significant difference in requirement of >1 day of apheresis between patients given Neupogen^®^ and Grastofil^®^ (42.1% vs. 36.8%, respectively, *p* = 0.67). 

**Figure 1:**
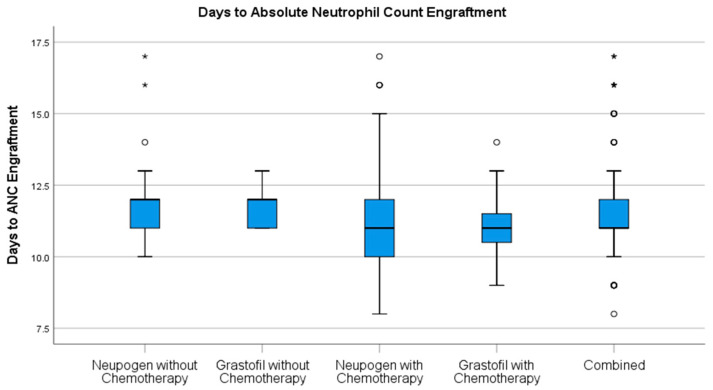
Days to ANC engraftment comparison for patients receiving Neupogen^®^ or Grastofil^®^. * denotes extreme outlier.

**Figure 2:**
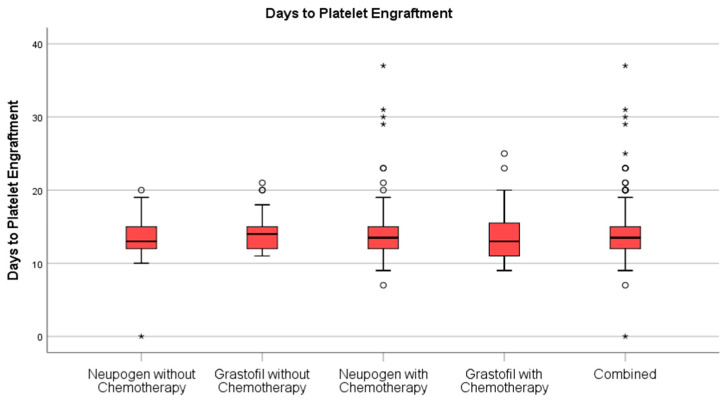
Days to platelet engraftment comparison for patients receiving Neupogen^®^ or Grastofil^®^. * denotes extreme outlier.

Stem cell engraftment (Table 2), described as ANC recovery (Figure 1) and platelet recovery (Figure 2) were highly similar between mobilization regimens.

**Figure 3:**
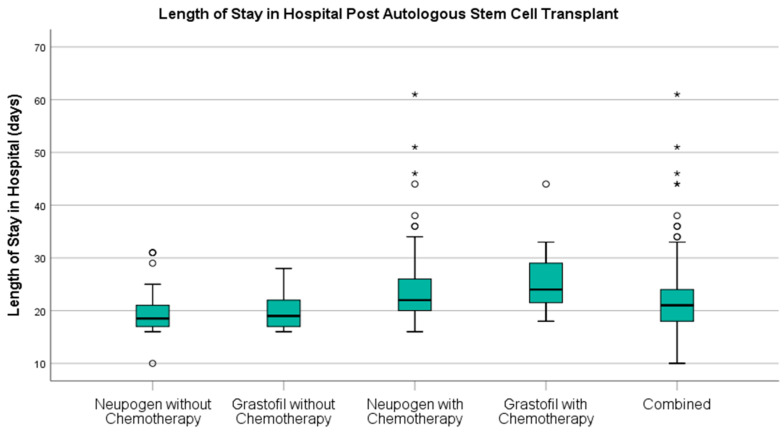
Length of stay in hospital for patients post autologous stem cell transplant. * denotes extreme outlier

There was no significant difference in length of stay in hospital (Figure 3) between patients who had received Neupogen and those who had received Grastofil^®^ (22.4 vs. 22.3 days, respectively, *p* = 0.82). Furthermore, there was no difference in hospital stay between patients with similar diagnoses, independent of the inclusion of chemotherapy as part of their mobilization. Multiple myeloma patients who received Neupogen^®^ had a median length of stay of 18.5 days (IQR = 17.0–21.0) compared to Grastofil^®^ median length of stay 19.0 days (IQR = 17.0–22.0) (*p* = 0.75). Likewise, patients diagnosed with lymphomas or other malignancies who received chemotherapy concurrently with G-CSF had similar lengths of hospital stay irrespective of receiving Neupogen^®^ (median = 23.0, IQR = 21.0–27.5) or Grastofil^®^ (median = 25.0, IQR = 23.0–29.0) (*p* = 0.24).

## 4. Discussion

This study presents a real-world experience of ASCT mobilization and engraftment results from 217 patients with multiple myeloma, amyloidosis, lymphomas, and other hematological malignancies treated with originator filgrastim or biosimilar filgrastim. This study is notable for the total number of patients included as well as the diversity of diagnoses reflecting the community clinical practice setting. To the best of our knowledge, this comparative study represents one the first real-world evidence studies using biosimilar G-CSF in the ASCT setting in Canada. In determining the effectiveness of biosimilar filgrastim for hematopoietic stem cell mobilization prior to ASCT, we observed no difference in the primary endpoint of CD34+ mobilization and collection yields between the two agents. Additionally, no clinical differences were detected in successful engraftment as demonstrated by neutrophil and platelet engraftment or in the overall length of hospitalization post-transplant. These findings build upon other real-world evaluations of biosimilar G-CSF products in the ASCT mobilization setting to help extend global evidence supporting confidence in using biosimilar G-CSF across all approved indications [14,18,19,20]. Furthermore, these results should alleviate clinician concerns about the effectiveness of biosimilars across indications, particularly those indications which are considered to have been granted by extrapolation. 

In addition to successful mobilization, no statistically significant differences were detected in collection outcomes such as number of apheresis, and adjunctive Plerixafor use, or in engraftment kinetics; observations that collectively reflect both clinical similarity and cost-neutrality in terms of pharmacologic and logistical resource requirements for patients undergoing ASCT. Unlike some other comparisons of biosimilar and originator G-CSF in the setting of ASCT mobilization which utilized Plerixafor for all patients, we found that only 23.4% of Grastofil^®^ patients, and 25.9% of Neupogen patients required Plerixafor to boost CD34+ count prior to apheresis which demonstrates a significant resource savings [18,21]. In this study, we observed Plerixafor use to be marginally higher among patients with lymphomas and other malignancies who received chemotherapy during mobilization, with no significant difference between G-CSF cohorts. Neutrophil engraftment, platelet engraftment, and length of stay in hospital were all similar between groups and consistent with clinician expectations. The lack of clinical differences observed, combined with the prospect of reduced financial burden, should be compelling in support of the continued adoption of biosimilar G-CSF across other provinces where biosimilar conversion has been lagging.

While randomization typically mitigates any potential differences between treatment groups, this retrospective design is well suited to analyzing two treatment populations at a pre- and post- practice change timeframe. Across the two time periods, there were no significant changes in the mobilization algorithm practiced at this institution. Sample sizes were robust and patient characteristics were fairly consistent between groups. Although mean age of patients was slightly higher among Grastofil^®^ mobilized patients than Neupogen^®^ patients (59.3 vs. 55.9, respectively), this difference did not appear to have a clinically meaningful impact. There were no significant differences between groups with regards to gender or diagnoses, however, a significantly higher proportion of patients mobilized with Neupogen^®^ were also concurrently treated with chemotherapy versus the more recently treated Grastofil^®^ patient population. Ideally, analyzing a more homogenous patient cohort with all patients receiving the same chemomobilization regimen is recommended to enhance the clinical comparability exercise for biosimilars; however, this would limit translating findings to a more diverse patient population and we view the collective of hematological malignancies reflected in our cohorts as a strength [22]. 

Additionally, this study describes univariate comparisons across each of the patient factors captured, and we did not see the need to develop a multivariate model, because each predictive variable that has been previously described to influence mobilization actually favored the historic comparator arm. Most notably, advanced age and extensive chemotherapy are considered risk factors for poor mobilization. However, it has been well documented that chemomobilization in appropriate candidates, typically with cyclophosphamide, is actually associated with higher CD34+ yield, lower failure rate, and improved engraftment kinetics [23]. In this data set, the biosimilar arm demonstrated higher age and more frequent G-CSF single agent mobilization, both negative predictors for successful mobilization, but was still numerically favorable on several outcomes, with no significant differences detected in any endpoint.

We did not evaluate the data set for safety outcomes associated either with the G-CSF agent itself or during the at-risk period prior to engraftment such as rate of FN, antibiotic requirement, or additional health care resource needs. Total length of hospitalization was similar between groups, but does not adequately describe complications which may occur during that time. Despite the lack of data regarding safety outcomes, the investigators did not note any overall differences in the rates of common adverse events during ASCT recovery. 

This study was intended to evaluate mobilization, collection, and subsequent engraftment outcomes in the ASCT setting following the implementation of a biosimilar G-CSF in place of the originator brand after approval by the regulatory authority and subsequent market availability. This single institution evaluation was conducted in the second year following formulary change and the number of patients mobilized with biosimilar filgrastim was limited in comparison to available historic comparator data. This study is limited by its retrospective design and small Grastofil^®^ sample size. Nevertheless, the institution and the investigators were motivated to analyze the available data to substantiate the adoption of an alternative G-CSF agent. 

Collectively, these data expand upon the body of clinical evidence to include a labeled indication of use that was not submitted to the regulatory authority as part of the biosimilar totality of evidence. Approval of biosimilar indications relies upon a tailored regulatory pathway critical to lowering developmental costs to produce lower priced products, which can be coupled with increased competition for availability of less expensive alternatives. Furthermore, the increasing acquisition cost of biologic medicines, particularly evident in the need to reduce the escalating cost of cancer treatment, provides an opportunity for biosimilars to impart greater value to healthcare while most importantly also ensuring similar outcomes [24,25,26]. The regulatory pathway for biosimilars is predicated upon a stepwise process in which biosimilars demonstrate similarity in all critical aspects of the drug, including confirmatory clinical efficacy studies conducted in the most sensitive patient population to reduce uncertainty in concluding biosimilarity [27]. The approval in a specific indication without the need for a clinical trial is based upon the totality of an evidence approach and is dependent upon clinical appropriateness, particularly similarity of mechanism of action and comparable risk profile across different indications.

## 5. Conclusions

Overall, the findings of this study together with the experience presented by other investigators on the clinical efficacy and safety of biosimilar filgrastim in ASCT should serve to reassure clinicians, other healthcare professionals (HCPs), and patients that the ability to extrapolate to an authorized indication of the reference product and its regulatory application for biosimilar development derives from robust evidentiary principles and sound justification. When comparing the use of biosimilar or originator G-CSF based mobilization regimens in terms of important ASCT outcomes, including stem cell harvest success, Plerixafor^®^ use, more than one apheresis day being required, the time to engraftment, and the length of stay in a hospital, no significant difference was found, thereby indicating that both products have similar efficacy. The use of biosimilars can provide substantial cost savings to the health care system while delivering comparable efficacy outcomes. In Saskatchewan, the implementation of biosimilars required engagement, education and collaboration of all members of the health care team. Recognition and frequent communication of the cost savings and re-investment into patient care has allowed for a smooth adoption.

## Figures and Tables

**Table 1:** Demographics/ Descriptive Statistics.

Characteristic	Neupogen^®^	Grastofil^®^	*p*-Value
	*n* = 170	*n* = 47	
Demographics			
Age, mean (STD)	55.9 (10.5)	59.3 (7.3)	0.048
>60 yrs.	67 (39.4%)	25 (53.2%)	0.09
Gender, male	106 (62.4%)	33 (70.2%)	0.32
Diagnosis			0.6
MM/Amyloid *	94 (55.3%)	28 (59.6%)	
Lymphoma/other **	76 (44.7%)	19 (40.4%)	
Mobilization Regimen			0.02
G-CSF only	64 (37.6%)	26 (55.3%)	
Concurrent Chemotherapy	106 (62.4%)	21 (44.7%)	
Concurrent Chemotherapy regimen			0.024
CE	76 (44.7%)	19 (40.4%)	
Cyclophosphamide	30 (17.6%)	2 (4.3%)	

* Multiple myeloma 117, Amyloidosis 4, MM/Amyloid 1; ** Hodgkin’s disease (HD) 18, Mantle cell lymphoma (MCL) 17, Diffuse large B-cell Lymphoma (DLBCL) 16, Follicular lymphoma (FL) 8, Double-hit lymphoma (DHL) 6, Central nervous system (CNS) lymphoma 6, T-cell lymphoma 3, Anaplastic large T-cell lymphoma (ALTCL) 3, Peripheral T-cell lymphoma (PTCL) 2, Marginal zone lymphoma (MZL) 1, MZL/MCL 1, Transformed 13, Other 1.

**Table 2:** Mobilization and Engraftment Outcomes.

Outcome	Neupogen^®^	Grastofil^®^	*p*-Value
	*n* = 170	*n* = 47	
Mobilization			
Successful ASCT Harvest	157 (92%)	47 (100%)	0.075
CD34+ Harvested, mean (StD)	6.1 (3.3)	6.5 (3.9)	0.88
Plerixafor Doses	44 (25.9%)	11 (23.4%)	0.72
Number of Apheresis			
1	87 (52.2%)	19 (40.4%)	0.19
2 to 3	83 (48.8%)	28 (59.6%)	
Engraftment			
Days to ANC Recovery, mean (StD)	11.6 (1.6)	11.6 (1.0)	0.51
Days to Platelet Recovery, mean (StD)	14.0 (4.0)	14.2 (3.6)	0.65
Total Length of Stay, mean (StD)	22.4 (6.6)	22.3 (5.5)	0.82

## Data Availability

The data presented in this study are available in this article.
